# Royal Veterinary College

**Published:** 1892-01

**Authors:** 


### SELECTIONS.

### THE ROYAL VETERINARY COLLEGE.*

### A Sketch of Its Progress.—II., 1817 to 1839.

During the first twenty-five years of this century the College passed a very uneventful existence—turning out students with a certificate, increasing its reputation as a teaching institution, and slowly building up a veterinary science to guide the art. Although the staff added nothing to our literature. some of its graduates did. Blaine published his "Veterinary Outlines," Percivall his "Lectures," Bracy Clark wrote on the horse's foot, and White produced a work on "Farriery," which went through a number of editions. Professor Coleman wrote nothing after his book on the horse's foot, and we do not know any special work or research of a scientific nature initiated by him at the College. The Assistant Professor Sewell seems to have done a little more. He introduced the operation of neurotomy for incurable foot lameness, and deserves credit for its practical adoption, although it was not an original discovery. Moorcroft practised the operation in 1800, and one of the elder Fields long afterwards mentioned his participation in the operation. Navicular disease was recognized as a cause of foot lameness, and Coleman had, previous to 1816, added to the College Museum a specimen of a diseased bone. To Turner, however, belongs the credit of discovering that the disease was the common cause of chronic foot lameness. He wrote a paper on the subject as early as 1816, at which time Coleman looked upon it as a rare condition, and believed the well-known symptoms were due to "contraction." To Assistant Professor Sewell we are indebted for the operation of peri-osteotomy, which he recommended in the treatment of splints. For the use of sulphate of copper as a remedy (then called cure) for glanders, the Professor obtained the special thanks of the Governors, and a pecuniary reward, which was not undeserved; for although he had not, as was at first thought, discovered a cure, he had undoubtedly carried out a careful series of experiments. In 1816 Sewell visited the Continental veterinary schools, and submitted to the Governors of the College a report on his travels. The report certainly does little credit to his powers of observation, being a mere bald catalogue of the stables and buildings and horse-

* *The Veterinary Record*, November, 1891.

shoes which he saw. Not a single note worth remembering, bearing upon veterinary science or teaching, is to be found in it. If Sewell's Continental tour taught him anything, it was perhaps the use of setons, which he employed in practice to a very large extent. A very favorite application of the seton in the College practice of his time was the insertion of one through the frog for foot lameness—a practice which was continued down to recent times by the oldest living practitioners.

In 1825 the teaching staff of the College was increased by the appointment, as Assistant Demonstrator of Anatomy, of Mr. Richard Vines, who had joined the Institution as a student the previous year. Vines appears to have been an honest worker, with rather more taste for physiology than anatomy. His writings indicate a desire for scientific inquiry somewhat beyond his intellectual capacity, and his chief work, "On the Blood," is a curious collection of hypotheses sententiously stated, but based on very superficial observations.

In 1826 Mr. F. Cupiss, of "Constitutional Ball" notoriety, retired from the office of Clerk and Dispenser of the College, and was succeeded by Mr. J. T. Morton, who had, after serving his apprenticeship to a chemist and druggist in Devonshire, held a situation in the house of a manufacturing and wholesale druggist in the city. Marton very soon evinced considerable interest in the action of medicines upon the horse, and obtained leave to administer drugs occasionally, with the direct object of noting their action. Full proof of his perseverence in this direction is said to be preserved in the College archives, in the form of manuscript notes, filling three large folio volumes.

When we arrive at the year 1828 we reach more certain data as to the College and its graduates. In this year two monthly periodicals made their appearance—*The Farrier and Naturalist* and *The Veterinarian*. The former only lasted two years, and was conducted with great rancour in open opposition to the College. The undisguised contempt with which it spoke of the Institution, and the undignified and scurrilous language which it applied to all its antagonists render it a very untrustworthy guide to the events which it chronicles.

The *Veterinarian* was a very different journal, and although opposed to some of the methods of the College, its objections were stated fairly, the reforms it desired were announced in temperate language. There can be no doubt that at this time the College had failed to give satisfaction to the veterinary practitioners whom it had educated. Coleman had lost his early enthusiasm, and illness often incapacitated him from work. He failed to appreciate the advances made by leading practitioners who had long left the College, and he declined to see that the standard of tuition given thirty or forty years ago had become insufficient. Coleman was an autocrat and conducted the College exactly as he thought fit The Governors trusted him implicitly, and his friend, Sir Astley Cooper —chief of the Veterinary Examining Board—supported him in the most faithful manner whether he were right or wrong. The great source of disagreement between the College and the profession arose over the Examining Board, which consisted entirely of medical men, assisted by Professors Coleman and Sewell. Meetings were held, interviews with Coleman took place, and deputations waited upon the Governors. Complaints were made that students after only five or six months at College were granted a certificate by the medical examiners and sent out to practice. It was stated that such ill-taught men brought discredit upon the profession, and that a longer course of study shonld be insisted upon, and that some practitioners of the veterinary art should be added to the board of doctors who decided that candidates were fit to practice. Coleman pretended to accept the proposed reforms, but the Governors of the College referred the matter to the Examining Board, who declined to act with any veterinary examiners. A proposal was made that the board should consist of two sections—medical and veterinary—which should examine separately, but the Governors very properly declined to accept this arrangement as impracticable ; still they gave no countenance to the very practical and needful alternative of a board of joint medical and veterinary examiners. The College authorities clearly looked upon the proposed reference as an attempt on the part of a section of practitioners to intrude upon their sphere, and to interfere with the arrangements of the Institution. The practitioners honestly believed that the profession was being injured by the methods of the College, and it was more than suspected that the opposition to reform was due entirely to Coleman, backed by Sir Astley Cooper. The agitation did some good ; a promise was made that no student should be examined until after, at least, one year's study. Within the College no improvement was made in the teaching, but the efforts of extra-mural teachers were not discouraged. Youatt held classes in his school in Nassau street, where students might attend lectures on the anatomy, physiology, and pathology of the ox, sheep, and dog. Charles Spooner opened a school of anatomy in premises nearly opposite the College about

1830, and was shortly after joined by Morton who lectured on pharmacy and materia-medica. To these men and to W. Percivall, who published the first systematic treatise in English on the *Anatomy of the Horse*, the veterinary students in "the thirties" owed a deep debt of gratitude. The teaching of veterinarians outside of the College walls, combined with the classes gratuitously opened by medical teachers to veterinary students, enabled young men to attain considerable proficiency, in spite of the utter neglect of complete and systematic instruction within the College. The disagreement between the practitioners and the College subsided, but was not extinguished. The evils of insufficient teaching and defective examination were felt and acknowledged more deeply and widely by the profession as the active oppositition to the College became less pronounced. and the first hint of real professional independence is to be found in a letter to *The Veterniarian* in 1830. "An old Veterniarian" writes proposing to memorialise Government to establish a Royal College of Veterinary Surgeons independent of any school. This, he argues, would leave the St. Pancras school to arrange its own methods, whilst the profession could then protect itself against the ignorance which discredited its members, as the examination would then be strictly *veterniary*. This seems to have been the original germ from which issued the Charter of 1844.

In 1834 the total number of veterinary surgeons on the published list was 624, and the number of students at the College had increased from thirteen at the time of Professor Coleman's appointment, to about seventy.

In 1835 Coleman was presented with his bust in marble, and at the ceremony and the dinner which followed peace and good-will seemed to reign supreme. The students were no whit more enthusiastic and generous than were the practitioners, who came from all quarters to honor their venerable and respected teacher. In the following year Professor Sewell, who had then been a devoted follower and colleague of Coleman for forty years, was presented by the members of the London Veterinary Medical Society with a service of plate ; Coleman made the presentation and Sewell replied, both the Professors referring to "the cordiality of feeling and unity of purpose which existed between them."

Nothing eventful is recorded now until 1838, when the English Agricultural Society was founded under the patronage of the greatest and richest in the land. Amongst the "resolutions and purposes" of the Society was one "to take measures for improving the veterinary art as applied to cattle, sheep and pigs." On December 18, at the half-yearly meeting of the Society, a passage in the report was as follows: "Application has been made to the Governors of the Veterinary College, stating our wish to assist in directing attention to the diseases of cattle and sheep by contributing from our funds." About six months later *The Veterinarian* announces that "the English Agricultural Society and the Governors of the College, after various interviews and much opposition from *one quarter* (Coleman?), have agreed to provide for the study of other animals than the horse." This is the first indication of progress made by the school in deference to outside suggestion.

In an editorial, to be found in *The Veterinarian* of July 1, 1839, we read: "During a considerable portion of the season just closing both the Professors have been seriously indisposed. Week after week not a lecture was given in the theatre, nor would there have been one demonstration in the dissecting room had not Mr. Spooner fortunately resided near the College, and, at the request of Professor Sewell, devoted the whole of his time to the anatomical instruction of the pupils and the treatment of the patients." This *indisposition* in Professor Coleman's case terminated fatally, and the veteran died on July 14, before any effect could be given to the proposed reforms.

Undoubtedly Coleman ruled the College as an actocrat, and was personally responsible for its successes and its failures. His last twenty years of office contributed little, if anything, to veterinary science or art, but much may be forgiven him for the good work he did in his earlier days. He directed his efforts chiefly to the social aspect of the profession, and was not unsuccessful.

(*To be continued.*)

